# Genetic susceptibility to depressive symptoms in middle-aged to older Americans: time-varying effects and effect modification by early psychosocial factors

**DOI:** 10.1007/s00127-025-02987-0

**Published:** 2025-08-30

**Authors:** Hannah E. Wilding, Walter G. Dyer, Brianna Sutara, Sung-Ha Lee, Ashley N. Linden-Carmichael, Stephanie T. Lanza, Harold H. Lee

**Affiliations:** 1 The Penn State College of Medicine, Hershey, PA 17033 USA; 2 Department of Psychology, The Pennsylvania State University, University Park, PA 16802 USA; 3 Department of Biology, The Pennsylvania State University, University Park, PA 16802 USA; 4 Department of Psychology, Yonsei University, Seoul, 03722 South Korea; 5 Department of Counseling Psychology and Human Services, University of Oregon, Eugene, OR 97403 USA; 6 Department of Biobehavioral Health, The Pennsylvania State University, University Park, PA 16802 USA

**Keywords:** Depression, Polygenic risk scores, Early psychosocial factors, Childhood stress, Aging

## Abstract

**Purpose:**

We examined age-varying genetic influences on depression across young adulthood to older adulthood and the moderating role of early psychosocial factors.

**Methods:**

Data are from the Health and Retirement Study (HRS) with 6,977 European Americans (57% women) from 2006 to 2016 (*M* age 62.4 ± 14.3, range 26–101 years in 2006). The polygenic score (PGS) for major depression was operationalized as a binary variable at the 75th percentile. Early psychosocial factors examined included maternal warmth, parental education, perceived financial status, and childhood stressful events. Depressive symptoms were measured by the Center for Epidemiological Studies Depression Scale (CES-D; range: 0–8). We utilized time-varying effect modeling to determine the survey wave when genetic risk most affected depressive symptoms. Within this wave, we analyzed the age-varying effect of genetic risk on depressive symptoms and conducted interaction analyses between PGS with each early psychosocial factor.

**Results:**

The wave-varying effect model revealed that the genetic effect was strongest in 2006. During that year, genetic effects remained significant and stable across age groups, from middle-aged to older adults. In 2006, without negative experiences, those at high genetic risk for depression had 51–60% higher odds of depressive symptoms (CES-D ≥ 3). Conversely, without genetic risk, adverse early psychosocial factors raised depression risk by 37–54%. No multiplicative or additive interaction was observed between genetic risk and psychosocial factors.

**Conclusion:**

Identifying individuals with higher genetic susceptibility and adverse early experiences may inform targeted preventive approaches.

**Supplementary Information:**

The online version contains supplementary material available at 10.1007/s00127-025-02987-0.

## Introduction

Major Depressive Disorder (MDD) is characterized by acute and chronic symptoms of sadness, anhedonia, guilt, decreased energy, concentration difficulties, sleep disturbances, and suicidal ideation [1]. This escalating public health concern affects approximately 300 million individuals worldwide [2], with an economic burden of $382.4 billion annually in the United States [3–5]. Middle-aged adults experience an increased susceptibility to and strain of mental disorders [6], and the prevalence of depressive symptoms in older adults is 32% [7]. Despite ongoing efforts, the pathogenesis of MDD remains incompletely elucidated.

Individuals who have first-degree relatives with MDD are nearly three times more likely to develop MDD themselves [8], and twin studies suggest the heritability of MDD to be about 31–42% [8, 9]. Genome-wide association studies (GWAS) have delineated the multifocal genetic nature of depression. A recent GWAS meta-analysis of 480,359 individuals identified genetic variants associated with MDD [10]. The identified genetic variants can then be accumulated into a single index of genetic risk for the phenotype, polygenic scores (PGSs). In recent years, advanced PGSs, which optimize scores to maximize their explanatory power regarding phenotypic variance [11–13], have explained a non-trivial proportion of phenotypic variance, distinguishing them from earlier studies that assessed genetic risk based on phenotypes associated with single nucleotide polymorphisms (SNPs) or PGSs derived from limited SNP sets (5-100 SNPs). A key methodological advantage of these PGSs lies in their enhanced statistical power to detect interactions between genetic and environmental factors.

Using the contemporary PGSs, epidemiological research on depression examining interactions between genetic and psychosocial factors has swiftly expanded in the past five years [14–20]. A substantial number of individuals exhibiting depressive symptoms without formal diagnosis paradoxically contribute more to the disease’s prevalence, termed the “prevention paradox [21]. Despite the public health relevance of investigating the moderately depressed population, most existing studies primarily focus on high-risk populations, such as individuals diagnosed with clinical depression. Among genetic epidemiological research focused on depressive symptoms, only a few examined how early psychosocial factors—another strong determinant of depressive symptoms [22, 23]—exacerbate or buffer the genetic influence on depressive symptoms later in life [19, 20]. For example, in a study of 718 Chinese adolescents aged 10–14 years, PGS for depression and childhood trauma were associated with heightened depressive symptoms, yet the interaction was not statistically significant [20]. Similarly, no significant interaction between PGS for depression and peer victimization was found in a study utilizing data from 748 participants in the Quebec Longitudinal Study of Child Development [19]. In both studies, the authors acknowledged the limitation of small sample sizes that constrain the power for interaction analyses. Another plausible explanation for the nonsignificant findings is that the ages during which genetics exerts its effect may differ. Nascent literature on PGS has shown that the magnitude of genetic impact on phenotypes varies across different stages of life [24–26].

To address this gap, this study investigated (1) the time-varying effects of PGS on depressive symptoms and (2) the ways in which early psychosocial factors modify genetic influences on depressive symptoms. We used time-varying effect modeling (TVEM) [27] to assess depressive symptoms as a function of polygenic scores across different ages. During the period when the genetic effect was strongest, we examined whether early psychosocial factors influence genetic predisposition to depression in middle-aged and older adults, using data from the Health and Retirement Study (HRS).

## Methods

### Study sample

The HRS is a nationally representative, longitudinal panel study in the US, created to gather extensive health and behavioral information for individuals aged 50 years and older. Surveys are collected from approximately 20,000 participants every two years and a new cohort is included every six years [28]. Out of 15,190 randomly selected HRS participants chosen for additional in-person visits to provide genotype data, 6,977 European Americans provided outcome data on depressive symptoms during at least one wave between 2006 and 2016. The documentation report for the HRS data highly recommends that users perform analyses separately by ancestral group. We present findings only from the HRS European ancestry sample (*N* = 6,977; mean age in 2006 = 62.4 ± 14.3; age range 26–101 years; 57% female). The PGS in the present study is computed based on GWAS results primarily among individuals of European ancestry. This results in weaker predictive power in PGS derived from non-European populations due to variations in allele frequencies, linkage disequilibrium, and overall genetic structure across different ethnic groups [29–31]. Although we initially included African American participants, limited predictive accuracy and transferability of their PGS led to their exclusion. The study was deemed exempt by the Pennsylvania State University Institutional Review Board because the data were de-identified.

Of the 6,977 European Americans, the variables had low missingness (< 3%), except for maternal warmth (20%), parental education (12%), childhood stress events (11%), and CES-D measures (12–31%). For all missing covariates, we conducted multiple imputations (*n* = 5) using chained equations, a flexible method that also addresses attrition-related issues [32, 33]. This method tends to be more flexible than other methods for handling missing data and helps address problems associated with attrition [34, 35]. To enhance the precision of the imputation, we expanded the set of imputation variables to include socioeconomic indicators (e.g., total income, wealth, marital status), survey weight-related variables (e.g., sampling weights, stratification variables, respondent weights), and the complete set of 58 precomputed genetic risk scores, in addition to the primary study variables. We imputed data with the MICE package in R (version 4.4.2) and combined results using the MITOOL package in R [36].

### Measures

#### Depressive symptoms

Depressive symptoms were assessed using a modified eight-item Center for Epidemiologic Studies Depression Scale (CES-D) [37] every two years from 2006 to 2016. This self-report measure can identify individuals at a heightened risk for developing depression and has comparable psychometric properties to the twenty-item CES-D [37]. The CES-D score is the sum of six “negative” indicators minus two “positive” indicators. The negative indicators assessed whether participants experienced sentiments related to depression (e.g., “felt alone”), and the positive indicators asked whether the respondents “felt happy” or “enjoyed life”, all or most of the time over the previous week. After reverse scoring the positive indicators, the total possible score ranges from 0 to 8, with higher scores indicating more severe symptoms. In the HRS, the 8-item version of CES-D has high internal consistency (Cronbach α = 0.81–0.83), with sensitivity and specificity of 0.71 and 0.79, respectively [38]. For analyses investigating time-varying genetic effects on CES-D score, we used continuous variables. For the interaction analyses, we categorized the CES-D score (range: 0–8) into a binary variable to facilitate a clinically significant interpretation of the gene x environment. Consistent with prior HRS studies that examined CES-D [39], we employed a threshold of 3 or higher to denote clinically meaningful depressive symptoms, corresponding with the recommended cut-off value of 16 from the original CES-D scale [40].

#### Polygenic risk for major depressive disorder

The PGS of Major Depressive Disorder (MDD) was computed by the HRS team using PRSice software and summary statistics from the 2018 Psychiatric Genomics Consortium genome-wide association study of major depressive disorder [10]. PRSice calculates polygenic scores using a clumping and thresholding method, which reduces redundancy among genetic variants and tests multiple significance thresholds to find the most predictive score [41]. The PGS of MDD contains 1,340,536 single-nucleotide polymorphisms that overlap between the HRS genetic database and the GWAS meta-analysis [10]. The HRS PGSs have been normalized within European ancestry to conform to a standard normal curve [42]. This PGS of MDD was examined as a binary variable in the main text for enhanced interpretability in interaction analyses. Individuals in the upper 25th percentile of PGS of MDD were grouped as ‘high’ genetic risk, and all others were grouped as ‘low to moderate’ genetic risk for developing depressive symptoms.

#### Early psychosocial factors

We reviewed all early-life psychosocial measures available in the Harmonized HRS (Version C, 1992–2019, January 2022 release), a data set designed to promote methodological consistency across international HRS-sister studies (e.g., ELSA, SHARE, KLoSA). To support potential cross-national comparisons, we selected the Harmonized HRS over the RAND HRS and identified four key variables assessed in 2006 for inclusion: (1) maternal warmth, (2) perceived financial status, (3) number of childhood stress events, and (4) parental education. These four early psychosocial factors were each examined as binary variables for enhanced interpretability of interaction analyses.

1. Maternal warmth was dichotomized as low vs. moderate/high. This variable was calculated by summing the following three components: “how much the respondent agrees that they had a good relationship with their mother before age 18” (1 [strongly disagree] to 5 [strongly agree]), “how much time and attention the respondent’s mother gave them when they needed it before age 18” (1 [not at all] to 4 [a lot]), and “how much effort the respondent’s mother put into watching over and making sure the respondent had a good upbringing before age 18” (1 [not at all] to 4 [a lot]). The HRS team transformed the first item’s 5-point scale into a 4-point scale by placing the neutral response at 2.5 and adjusting the remaining responses so 1 indicated the most positive and 4 the most negative. A maternal warmth mean score (range: 1–4, median: 3) of lower than the bottom 25% was considered “low,” while all others were “moderate/high.”
2. Perceived financial status during childhood was dichotomized as high vs. normal. This variable represents the respondent’s self-rated family financial situation while growing up until 16. Respondents who rated their childhood financial situation as “poor” were categorized as “high,” while those who reported a financial situation of “pretty well off” or “about average” were categorized as “normal.”
3. The childhood stress events indicator assessed whether participants experienced any of the following stressful or adverse events before age 18: (1) whether the participant had encounters with law enforcement, (2) whether either of the participant’s parents drank alcohol or used drugs often enough to cause issues in the home, or (3) whether the participant experienced physical mistreatment from either parent prior to turning 18. Responses were dichotomized such that participants reporting one or more events were labeled as having “exposure to childhood stress,” while those reporting none were considered “unexposed.”
4. Parental education was classified as “low” if at least one parent completed 8 years or less of schooling, and “normal” if both parents completed more than 8 years of education, based on participant reports.

### Statistical analysis

To examine the time-varying effect model based on HRS study wave between binary PGS of MDD and continuous CES-D score, we used the TVEM macro in SAS v9.4 [43]. TVEM flexibly estimates the association between PGS of MDD and continuous CES-D score across continuous time (i.e., wave, age) such that the models allow the effect of the PGS of MDD to change over time. Unlike traditional linear regression, TVEMs do not assume a specific shape or rate of change over time. Instead, TVEMs flexibly model associations, permitting the effect of the PGS to fluctuate across different times. This dynamic association is represented graphically, where changes in the association are depicted by time-varying regression coefficients and their corresponding 95% CIs. Significant associations are identified for ages at which the 95% CI does not encompass zero. We included PGS as a time-varying predictor of CES-D scores, using time (wave) as the unit of measurement from 2006 to 2016. We accounted for the correlation of repeated CES-D assessments by utilizing the ‘cluster’ argument and specified a normal distribution for the continuous CES-D scores using the unpenalized B-splines method [27, 43, 44]. Once we identified the peak wave, we then examined the time-varying effect based on age of PGS of MDD on CES-D (age: 26–101 years), using the same methods described above. TVEM analyses were conducted across the five imputed datasets. We pooled the beta coefficients and standard errors from multiple time points within these datasets using Rubin’s rule [45]. This pooling allowed us to generate the TVEM figures for our analysis. In the TVEM analysis, survey weights were not used to avoid inflating parameter estimate variances, which could undermine statistical efficiency [46, 47]. This approach was suitable since our focus was on the relationship between PGS and CES-D scores rather than making broad population inferences.

To investigate both multiplicative and additive interactions between genetic exposure and early psychosocial factors, we used multivariate logistic regression models. These analyses were conducted separately for four early psychosocial factors. We also created a cumulative early psychosocial score computed by standardizing child financial stress, child stressful events, parental education (less than or equal to 8 years), and reverse-coded maternal warmth, and then summing these standardized scores. Cumulative scores are statistically responsive even in small sample sizes and do not rely on assumptions about the relative importance or interdependence of multiple risk factors [48]. The top 5% was considered a “high cumulative early stress” score. Principal components are commonly used in genetic epidemiology research to address issues related to population stratification—i.e., differences in genetic backgrounds, including racial/ethnic differences, among individuals in a study—and to capture underlying genetic variation within a study population, thereby reducing potential confounding effects in genetic association studies. We used five principal components as recommended in the HRS codebook for the polygenic risk score [49]. To enhance the precision of the effect size estimates, we also included sex, age, and age-squared as covariates in interaction analyses. In TVEM analyses, we controlled for five principal components and sex as time-invariant effects.

Consistent with recommendations for presenting interaction analyses [50], we calculated Odds Ratios (ORs) and 95% confidence intervals (CI) of both multiplicative and additive interaction [i.e., relative risk due to interaction (RERI)] as well as the ORs of genetic and psychosocial exposure respectively, controlling for gene x early psychosocial factor interaction and the covariates. *InteractionR* package in R was employed to compute RERI [51]. A RERI greater than zero indicated a positive deviation from additivity, and it was considered significant when the 95% CI did not include zero, with RERIs calculated using the delta method based on model-derived ORs. Controlling for the same covariates, we also present stratified analyses by both genetic and psychosocial exposures following the recommendations for interaction analyses report [50]. Given 4 multiple tests with the 4 psychosocial factors, we applied Bonferroni correction, with p-interaction of 0.0125(= 0.05/4 early psychosocial factors).

### Sensitivity analyses

We conducted several sensitivity analyses to validate the robustness of our findings. Instead of using a 25% cut point for the PGS of MDD, we used a 10% cut point to perform the same analyses: (1) the time-varying effect of PGS of MDD (top 10% vs. the rest) on depressive symptoms and (2) the interaction between PGS of MDD (top 10% vs. the rest) and early psychosocial factors on depressive symptoms. We conducted sensitivity analyses examining both genetic and early psychosocial factors as continuous variables while controlling for each other in one model. We also performed sensitivity analyses assessing parental education with a cut point of 12 years (vs. 8 years).

## Results

### Sample characteristics

The characteristics of participants, categorized by PGS of MDD (top 25% vs. the rest), are presented in Table 1. In the full analytic sample of 6,977 European Americans, the average age at baseline in 2006 was 62.4 ± 14.3 years, and females constituted 57% of the sample (*n* = 3,991). The mean CES-D score was 1.46 ± 1.9, with a median CES-D score of 1.0. The proportion of participants who reported they had experienced clinically relevant depressive symptoms (CES-D score ≥ 3) was 22% (*n* = 1525). Low maternal warmth was observed by 30% of participants, low perceived financial status by 24%, childhood stress events by 26%, and low parental education by 21%. The age distribution of the HRS sample is bimodal, with peaks at ages corresponding to the original HRS and AHEAD cohorts as well as subsequent younger cohorts added in later waves. (Figure A) [52]. Participants younger than 50 are included primarily because the study interviews the spouses or partners of age-eligible respondents, regardless of their age [52].

**Table 1:** Sociodemographic characteristics of European Americans from the health and retirement study (*n* = 6,977; 2006)^a^

	Normal genetic risk for major depressive disorder (PGS-MDD ≤ 75th percentile)	High genetic risk for major depressive disorder (PGS-MDD > 75th percentile)
Age, mean (standard deviation), years	62.6 (14.3)	61.8 (14.1)
Women (%)	3,018 (58)	973 (56)
CES-D score in 2006 (%)	1.34 (1.9)	1.82 (2.2)
Clinically depressive symptoms (CES-D≥3) (%)	1,028 (20)	498 (28.6)
Low maternal warmth (%)	1,538 (23)	532 (26)
Poor perceived childhood financial stress (%)	1,214 (23)	443 (26)
Experienced childhood stress events (%)	1,345 (26)	465 (27)
Low parental education (%)	1,003 (19)	451 (26)

^a^Imputed datasets (*n* = 5) were used. The variables had low missingness (< 3%), except for maternal warmth (20%), parental education (12%), childhood stress events (11%), and CES-D measures (29%)

### Time-varying effects of PGS of MDD (top 25% vs. the rest) on CES-D score

**Fig. 1:**
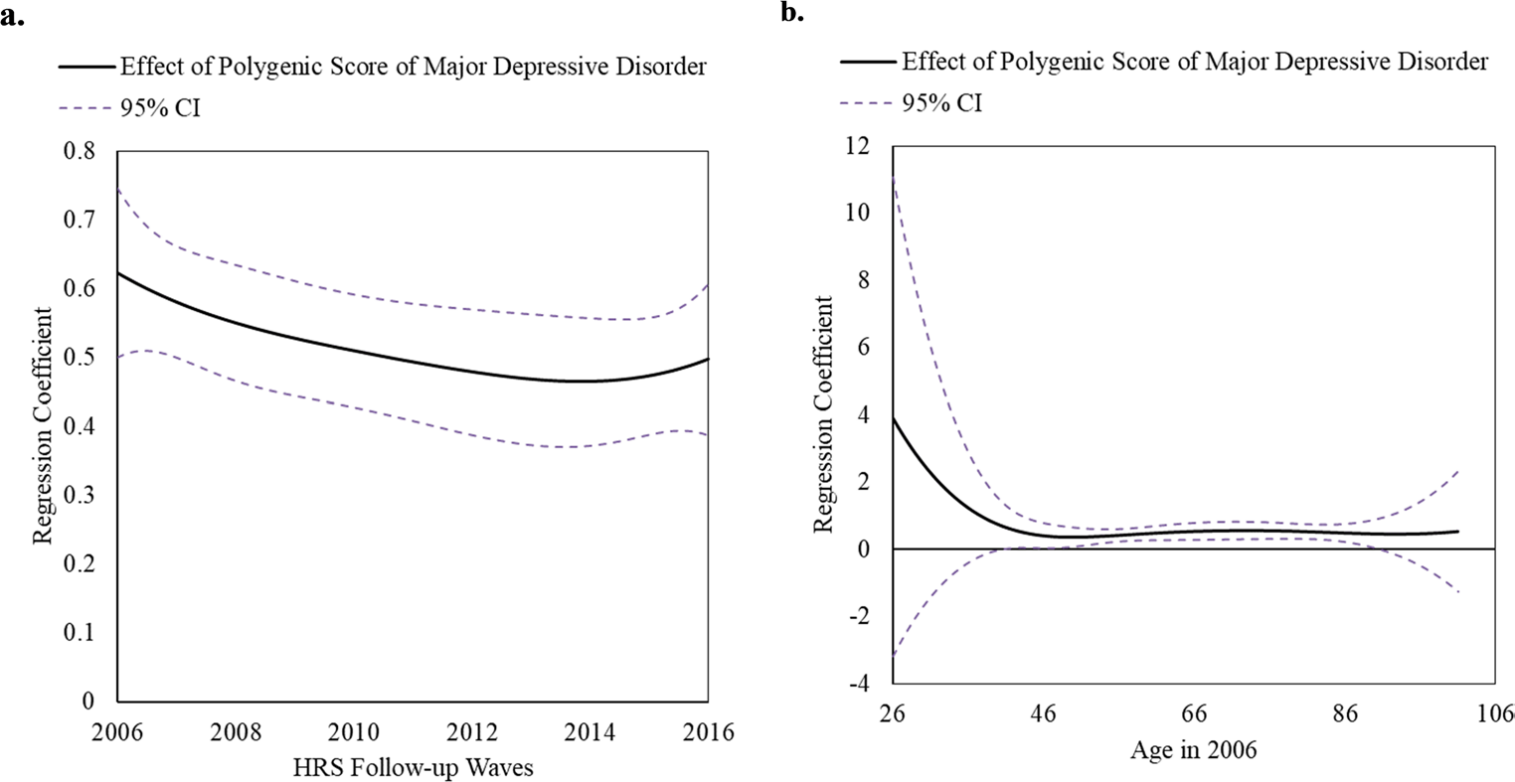
Time-Varying Effect of Polygenic Risk Score of Major Depressive Disorder (top 25% vs. the rest) on Continuous Depressive Symptoms (CES-D Score, range: 0-8) in the Health and Retirement Study (2006 to 2016, n=6,977, 62.4 ± 14.3 years in 2006). (**a**) Time-varying effect of genetic risk on depressive symptoms based on HRS study wave from 2006 to 2016. Effect was strongest in 2006. (**b**) Time-varying effect of genetic risk on depressive symptoms by different age (range: 26-100 years) in the 2006 wave HRS data

The time-varying effect model based on HRS study wave of PGS of MDD on continuous CES-D score has shown that the effect was the strongest in 2006 (Fig. 1a). In the 2006 wave, associations between PGS of MDD on CES-D were non-significant from ages 26 to 40 but significant and positive from ages 40 to 91 (Fig. 1b). Notably, only considering the trajectory of beta-coefficients, the time-varying effect model based on age revealed an L-shaped curve, with the genetic effect decreasing until mid 40 s, then stabilizing. Specifically, at age 41, individuals with a higher genetic risk for depression had a mean CES-D score that was 0.65 points higher (95% CI = 0.02–1.27) and maintained that level until 91, at which point genetic effect was no longer significant likely due to small number of participants around this age. However, the observation regarding the L-shape curve should be interpreted with caution, as the sample size was very small for ages under 41.

### Development of depressive symptoms: PGS of MDD (top 25% vs. the rest) and early psychosocial factors

Interaction analyses were employed using the 2006 wave, in which the PGS of MDD had the highest effect according to the TVEM analysis (Fig. 1a). Table 2 presents a comprehensive overview of (1) calculated ORs and corresponding 95% CI for both genetic and psychosocial exposures, accounting for their potential interactions, (2) stratified analyses by both genetic and psychosocial exposures, and (3) interaction analyses by the multiplicative scale and RERI.

**Table 2:** Interaction of polygenic score of major depressive disorder (top 25% vs. the rest) and early psychosocial experience on depressive symptoms (2006 CES-D score ≥3) in health and retirement participants (*n* = 6,977)

European Americans (6,977)	PGS-MDD ≤ 75th percentile (0)		PGS- MDD > 75th percentile (1)		OR^a^ of PGS-MDD within psychosocial strata	Interaction analyses
	N (cases/controls)	OR^a^ (95% CI)	N (cases/controls)	OR^a^ (95% CI)		
High maternal warmth (0)	667/3701 (18%)	1.00 (reference)	316/1206 (26%)	1.58 [1.26, 1.91]	1.58 [1.26, 1.91]	Multiplicative Scale: 1.06 [0.59, 1.53], *p* = 0.72 RERI: 0.34 [−0.22, 0.89] *p* = 0.24
Low maternal warmth (1)	357/1538 (23%)	1.37 [1.14, 1.59]	181/532 (34%)	2.28 [1.78, 2.79]	1.67 [1.29, 2.06]	
OR^a^ (95% CI) of the psychosocial factor within genetic strata		1.37 [1.14, 1.59]		1.44 [1.07, 1.81]		
Low childhood financial burden (0)	722/4025 (18%)	1.00 (reference)	337/1295 (26%)	1.55 [1.23, 1.87]	1.55 [1.23, 1.87]	Multiplicative Scale: 1.10 [0.57, 1.63], *p* = 0.61 RERI: 0.52 [−0.13, 1.17], *p* = 0.11
High childhood financial burden (1)	306/1214 (25%)	1.55 [1.24, 1.86]	161/443 (36%)	2.62 [2.01, 3.22]	1.69 [1.26, 2.13]	
OR^a^ (95% CI) of the psychosocial factor within genetic strata		1.54 [1.24, 1.86]		1.69 [1.24, 2.14]		
Low childhood stress events (0)	703/3894 (18%)	1.00 (reference)	340/1273 (27%)	1.61 [1.30, 1.91]	1.61 [1.30, 1.91]	Multiplicative Scale: 1.01 [0.52, 1.50], *p* = 0.97 RERI: 0.32 [−0.32, 0.97], *p* = 0.33
High childhood stress events (1)	324/1345 (24%)	1.52 [1.20, 1.83]	157/465 (34%)	2.45 [1.80, 3.10]	1.62 [1.17, 2.06]	
OR^a^ (95% CI) of the psychosocial factor within genetic strata		1.52 [1.20, 1.83]		1.52 [1.12, 1.92]		
High parental education (0)	777/4236 (18%)	1.00 (reference)	337/1287 (19%)	1.55 [1.29, 1.82]	1.55 [1.29, 1.82]	Multiplicative Scale: 1.06 [0.62, 1.50], *p* = 0.70 RERI: 0.42 [−0.17, 1.01], *p* = 0.16
Low parental education (1)	251/1003 (25%)	1.50 [1.23, 1.77]	161/1451 (36%)	2.47 [1.85, 3.10]	1.65 [1.21, 2.09]	
OR^a^ (95% CI) of the psychosocial factor within genetic strata		1.50 [1.23, 1.77]		1.59 [1.19, 1.99]		
Low cumulative early stressors (0)	965/5017 (19%)	1.00 (reference)	447/1625 (28%)	1.55 [1.31, 1.80]	1.55 [1.31, 1.80]	Multiplicative Scale: 1.41 [0.27, 2.56], *p* = 0.32 RERI: 1.36 [−0.27, 2.98], *p* = 0.10
High cumulative stress score (1)^b^	62/222 (28%)	1.62 [1.07, 2.18]	51/113 (45%)	3.53 [1.91, 5.15]	1.62 [1.07, 2.18]	
OR^a^ (95% CI) of the psychosocial factor within genetic strata		1.62 [1.07, 2.18]		2.27 [1.26, 3.28]		

OR = odds ratio; CI = confidence interval; PGS = polygenic score

N = number of subjects

Bold values represent statistically significant OR with *P* < 0.05

^a^ORs (95% CIs) were estimated by using logistic regression models with depressive symptoms (CESD≥3) as the outcome, adjusting for age, age-squared, sex, and 5 principal components

^b^The cumulative stress score was computed by standardizing child financial stress, child stressful events, parental education (less than or equal to 8 years), and reverse-coded parental warmth, and then summing these standardized scores. The top 5% was considered a high cumulative stress score

Unpacking the first row, maternal warmth, to enhance understanding of Table 2, the column titled *“OR of PGS-MDD within psychosocial strata”* illustrates how the impact of PGS of MDD (top 25% vs. the rest) on depressive symptoms varies by levels of psychosocial factors. For instance, among individuals with high maternal warmth, the odds ratio for depressive symptoms associated with PGS-MDD is 1.58 [95% CI = 1.26, 1.91]. This association is slightly stronger among those with low maternal warmth, with an odds ratio of 1.67 [95% CI = 1.29, 2.06], indicating that PGS of MDD (top 25% vs. the rest) may exert a greater influence in less supportive environments. Conversely, the row labeled *“OR (95% CI) of the psychosocial factor within genetic strata”* shows how the association between psychosocial factors and depressive symptoms differs by PGS of MDD (top 25% vs. the rest). For instance, among individuals within the low PGS-MDD group (below the 75th percentile), the odds ratio for depressive symptoms associated with low maternal warmth is 1.37 [95% CI = 1.14, 1.59]. This association is slightly stronger among those in the high PGS-MDD group (above the 75th percentile), with an odds ratio of 1.44 [95% CI = 1.07, 1.81]; again, these findings suggest that the negative impact of low maternal warmth on depressive symptoms may be amplified among individuals with elevated genetic vulnerability. Indeed, compared to those with no genetic risk and high maternal warmth, those with both high genetic risk and low maternal warmth had 2.28 [95% CI = 1.78, 2.79] odds of having depressive symptoms.

**Fig. 2:**
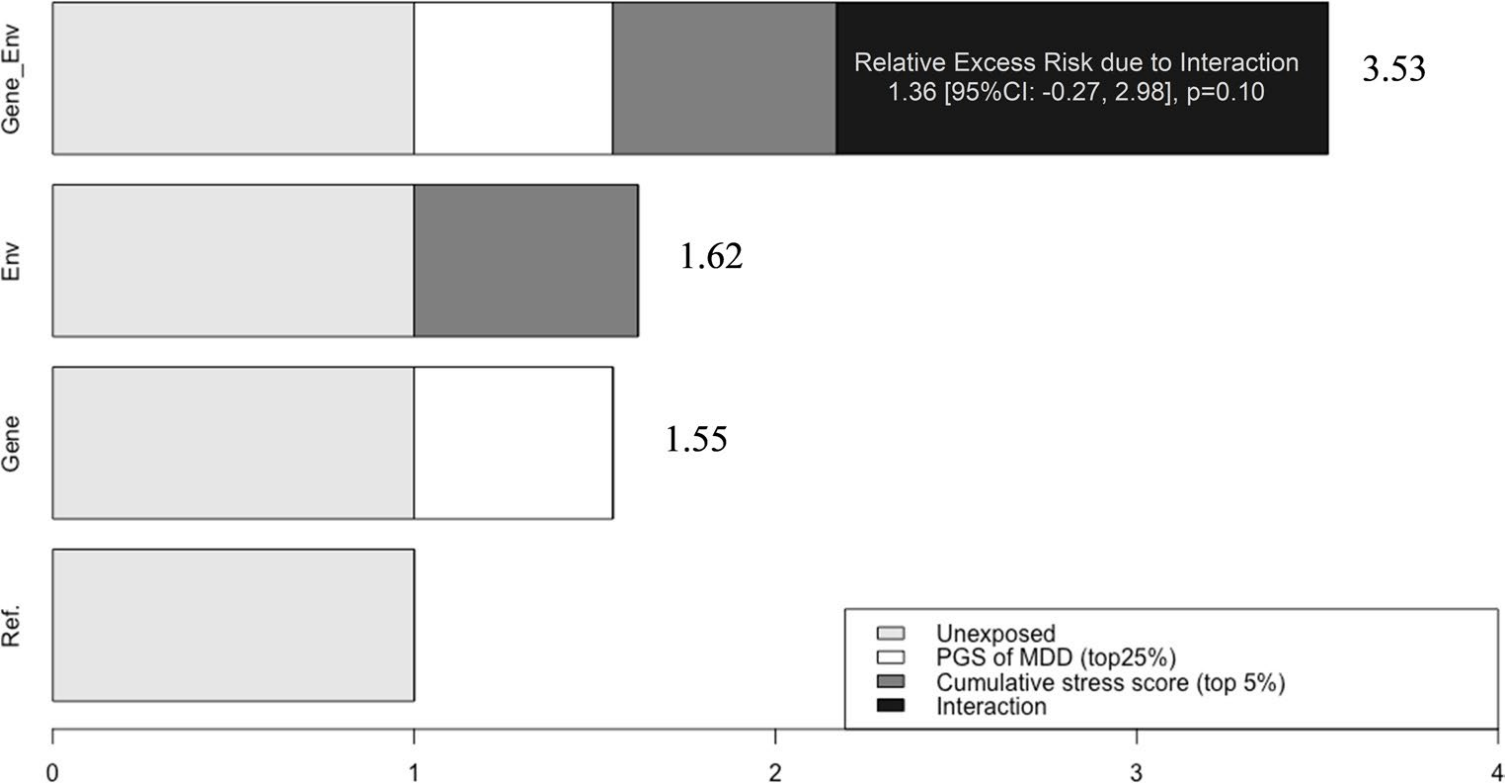
Interaction Effects of Cumulative Early Psychosocial Stress (top 5% vs. the rest) and Polygenic Score of Major Depressive Disorder (top 25% vs. the rest) on Depressive Symptoms (2006 CES-D Score ≥3) in European Americans (*n* = 6,977), Adjusted for Sex and Five Principal Components; Relative Excess Risk Due to Interaction measures additive interaction, which was not statistically significant (*p* = 0.10)

In the absence of negative psychosocial factors, individuals with a high genetic risk for depression exhibited greater odds of having depressive symptoms (CES-D≥3), with ORs spanning the range of 1.51 to 1.60 (Table 2). Conversely, in the absence of genetic risk, exposure to negative childhood experiences was associated with 1.37 to 1.54 times higher odds of depression, with high childhood financial burden demonstrating the strongest effect (OR = 1.54, 95% CI: 1.22–1.85). Although none of the individual early-life psychosocial factors significantly modified the genetic predisposition to depressive symptoms, the interaction between cumulative psychosocial adversity and genetic predisposition approached statistical significance. Independently, high genetic risk and cumulative early-life adversity were associated with 55% and 62% increased odds of depressive symptoms, respectively. When combined, they resulted in a 253% increase in odds, although the additive interaction was not statistically significant (RERI = 1.36, 95% CI = [−0.27, 2.98], *p* = 0.10) (Fig. 2).

Results from several sensitivity analyses showed a similar trend when we employed 10% (vs. 25%) cut point for the PGS of MDD (Figure B; Table A). That is, the effect of PGS on MDD tends to decrease with later waves or age, and all four psychosocial factors exacerbate the genetic predisposition to depressive symptoms.

### Sensitivity analyses

Sensitivity analyses examining both PGS of MDD and early psychosocial factors as continuous variables showed that PGS of MDD and each of the four early psychosocial factors were all statistically significant predictors of depressive symptoms when controlling for each other (Table B). The multiplicative interaction between cumulative early psychosocial score and PGS of MDD showed a trend towards statistical significance (p-interaction = 0.07) (Table C). In sensitivity analyses involving parental education with a cut point of 12 years (vs. 8 years), no statistically significant interaction was observed, consistent with the findings from the 8 years (Table D).

## Discussion

We investigated whether the genetic predisposition to depressive symptoms varies based on early psychosocial factors and age among ~ 7000 middle-aged to older Americans from the Health and Retirement Study who were followed from 2006 to 2016. The genetic effect was strongest in 2006. In 2006, the time-varying effect model based on age suggests that the genetic effect was statistically significant and stable from middle age to older adults (ages mid-40s to 90 s). Notably, the TVEM analysis by age appears to indicate that genetic effects are strong before age 40; however, these findings should be interpreted with caution, as the sample size was very small for ages under 41. Although individual early-life psychosocial factors did not significantly interact with genetic risk, the cumulative burden of psychosocial adversity showed a potential synergistic effect with genetic predisposition.

Our findings are consistent with results from previous research utilizing PGS to show independent associations between genetic and psychosocial factors with risk for depressive symptoms. In the study utilizing 748 participants in the Quebec Longitudinal Study of Child Development, data on peer victimization and genotype were collected for participants aged 12–13 years, and self-reported depressive symptoms were collected for participants aged 15–17 years. Peer victimization in early adolescence and PGS of depression were both independently associated with depressive symptoms in adolescence [19]. Likewise, in a study of 718 Chinese adolescents, data were collected on 10–14 year-old participants regarding history of childhood trauma, resilience, and depressive symptoms over a three year cohort study; PGS of depressive symptoms was also calculated. PGS of depressive symptoms and childhood trauma were risk factors for depressive symptoms, while the interaction between these terms had no significant association with depressive symptoms. Although the interaction analyses were not statistically significant in these two studies, both psychosocial factors and genetic factors were independently predictive of depressive symptoms, underscoring their distinct roles as observed in the present study.

While no single early-life psychosocial factor significantly modified the genetic risk for depressive symptoms, all four early psychosocial factors predicted depressive symptoms in adulthood, controlling for each other as well as for genetic risk. This observation is comparable to prior studies. For example, in a study of 1,988 participants followed from early adolescence to young adulthood, maternal warmth in young adulthood was shown to be protective of young adult depression [53]. Similarly, in a study of 2,257 participants, maternal warmth was shown to have an indirect effect on depressive symptoms and obesity, mediated by the participant’s conscientiousness. While prior research delineating the impact of perceived financial stress on depressive symptoms is sparse, one systematic review reported that a positive relationship between financial stress and depression was found in both high-income and low/middle-income countries [54, 55]. Our results corroborate and expand upon the findings of prior literature showing that the impact of early experiences can linger, affecting people into older age, highlighting the importance of developmental processes in influencing later-life depressive symptoms [56–58].

Although no single early-life psychosocial factor significantly moderated the genetic risk for depressive symptoms, the interaction between cumulative adversity and genetic predisposition approached significance, aligning with previous research. Previous HRS investigations have shown an interaction between genetic risk for depression and psychosocial experiences during adulthood (e.g., stressful life events) [59, 60]. Our study extends prior findings by demonstrating that early psychosocial factors exacerbate genetic predisposition to depression. Moreover, emerging literature indicates that the effect of genetics on phenotypes varies across different life stages [24–26]. To our knowledge, our findings are the first to report a time-varying moderation of depressive symptoms by PGS. Future research should replicate this in a diverse population with longer life trajectories.

Our findings highlight the importance of early intervention in preventing depressive symptoms by improving identification of high-risk groups. As the cost for DNA sequencing (i.e., SNP microarray) reduces, proactive health risk evaluation beginning at the earliest stages of life becomes feasible. Intervention during childhood is developmentally effective and economically efficient [61, 62]. Early childhood programs emphasizing soft skill formation have shown results of decreased prevalence of chronic health diseases such as heart disease, stroke, and cancer; decreased criminal activity; and increased productivity and success later in life [63–65]. To effectively target high-risk populations for early interventions, public health initiatives aimed at early intervention for depression may consider incorporating assessments of early lis study haseriences alongside genetic risks.

There are some limitations. First, the study primarily focuses on European Americans, limiting the generalizability of findings to other populations. Future studies with diverse racial groups may further confirm the current findings. Second, the study was conducted in a single cohort without replication. Given that we utilized early psychosocial factors from the Harmonized dataset, which is specifically operationalized to facilitate comparisons among HRS sister studies, future research may focus on replicating our findings in HRS sister studies across other countries. Such studies should incorporate a broader age range and more diverse populations to enhance the generalizability and robustness of the results. We relied on pre-constructed PGS provided by the HRS, which prevented us from recalculating them using newer GWAS or advanced methods such as Bayesian regression and shrinkage. Future studies with access to individual-level HRS data may leverage updated and trans-ancestry GWAS summary statistics with updated PGS computation software to enhance predictive performance [66]. Finally, HRS provides data on relatedness among participants only with a restricted data agreement. Hence, we were unable to account for genetic relatability in our analyses. Despite these limitations, the study has notable strengths. With a substantial sample size of 6,977 European American participants, the study provides sufficient statistical power to detect meaningful interactions between genetic and psychosocial factors. Furthermore, the inclusion of various early psychosocial factors—such as maternal warmth, perceived financial status, and childhood stress events—provides a comprehensive understanding of how these elements interact with genetic predispositions.

## Conclusion

Our findings highlight the compounded risk posed by genetic predisposition and cumulative early-life adversity in shaping long-term mental health outcomes. Addressing multiple early-life stressors through targeted early interventions may be essential for reducing the broader public health burden of depression. To further refine our understanding and broaden the applicability of these insights, future research should encompass a more expansive age range and diverse populations.

## Supplementary Information

Below is the link to the electronic supplementary material.

Supplementary Material 1

## Data Availability

Data are available for public access and download via Health and Retirement Study (HRS). HRS is a nationally representative, longitudinal panel study in the US, created to gather extensive health and behavioral information for individuals aged 50 years and older.
