# Supplementary material for: Genetic susceptibility to depressive symptoms in middle-aged to older Americans: time-varying effects and effect modification by early psychosocial factors
Source: Soc Psychiatry Psychiatr Epidemiol. Author manuscript; Available in PMC 2026 Mar 24. (PMC13008129; doi:10.1007/s00127-025-02987-0)
Supplement: Supplementary file 1 [file NIHMS2150237-supplement-Supplement.docx]

Supplements: Table of Contents

Figure A. Age distribution across waves in the Health and Retirement Study (2006 to 2016, n=6,977).	2

Figure B. Time-Varying Effect of Polygenic Risk Score for Depressive Symptoms (top 10% vs. the rest) on Continuous Depressive Symptoms (CES-D Score, range: 0-8) in the Health and Retirement Study (2006 to 2016, n=6,977, 62.4 ± 14.3 years in 2006)	3

Table A. Interaction of Polygenic Risk Score for Major Depressive Disorder (top 10% vs. the rest) and Early Psychosocial Experience on Depressive Symptoms (average CES-D Score from 2006) in Health and Retirement Participants (n=6,977)	4

Table B. Sensitivity analyses without continuous variables. Impact of Continuous Polygenic Score and Continuous Early Psychosocial Factors on 2006 Continuous CESD Score in 6,977 Participants	5

Table C. Interaction Between Genetic Risk for Major Depressive Disorder and Cumulative Early Psychosocial Factors in Relation to CESD Score:	6

Table D. The parental education cut point is 12 years. Interaction of Polygenic Risk Score of Major Depressive Disorder (top 25% vs. the rest) and Early Psychosocial Experience on Depressive Symptoms (2006 CES-D Score >3) in Health and Retirement Participants (n=6,977)	7

# **Figure A**. Age distribution across waves in the Health and Retirement Study (2006 to 2016, n=6,977).

| **a.** | **b.** |
| --- | --- |

# **Figure B**. Time-Varying Effect of Polygenic Risk Score for Depressive Symptoms (top 10% vs. the rest) on Continuous Depressive Symptoms (CES-D Score, range: 0-8) in the Health and Retirement Study (2006 to 2016, n=6,977, 62.4 ± 14.3 years in 2006) 

(a) Time-varying effect of genetic risk on depressive symptoms based on HRS study wave from 2006 to 2016. Effect was strongest in 2006. (b) Time-varying effect of genetic risk on depressive symptoms by different age (range: 26-100 years) in the 2006 wave HRS data.

# **Table A.** Interaction of Polygenic Risk Score for Major Depressive Disorder (top 10% vs. the rest) and Early Psychosocial Experience on Depressive Symptoms (average CES-D Score from 2006) in Health and Retirement Participants (n=6,977)

|  | PRS-MDD < 10th percentile (0) |  | PRS- MDD > 10th percentile (1) |  | OR^a^ of PRS-dps within psychosocial strata | Interaction analyses |
| --- | --- | --- | --- | --- | --- | --- |
|  | N  (cases/controls) | OR^a^ (95% CI) | N (cases/controls) | OR^a^ (95% CI) |  |  |
| High parental warmth (0) | 882/4443 (20%) | 1.00 (reference) | 134/478 (28%) | 1.43 [1.00, 1.86] | 1.43 [1.00, 1.86] | Multiplicative Scale:  1.29 [0.64, 1.93], p<0.001 RERI:  0.68 [-0.28, 1.64], p=0.17 |
| Low parental warmth (1) | 452/1836 (25%) | 1.35 [1.14, 1.56] | 75/220 (34%) | 2.46 [1.64, 3.28] | 1.83 [1.15, 2.50] |  |
| OR^a^ (95% CI) of the psychosocial factor within genetic strata |  | 1.35 [1.14, 1.56] |  | 1.73 [0.96, 2.51] |  |  |
| Low childhood financial burden (0) | 946/4791 (19%) | 1.00 (reference) | 141/521 (27%) | 1.45 [1.03, 1.87] | 1.45 [1.03, 1.87] | Multiplicative Scale:  1.17 [0.59, 1.75], p<0.001 RERI:  0.61 [-0.40, 1.62], p=0.24 |
| High childhood financial burden (1) | 388/1488 (26%) | 1.55 [1.27, 1.84] | 68/177 (38%) | 2.62 [1.74, 3.49] | 1.69 [1.03, 2.35] |  |
| OR^a^ (95% CI) of the psychosocial factor within genetic strata |  | 1.55 [1.27, 1.84] |  | 1.81 [1.05, 2.57] |  |  |
| Low childhood stress events (0) | 922/4688 (20%) | 1.00 (reference) | 131/495 (27%) | 1.54 [1.16, 1.92] | 1.54 [1.16, 1.92] | Multiplicative Scale:  1.03 [0.52, 1.54], p<0.001 RERI:  0.32 [-0.64, 1.29], p=0.51 |
| High childhood stress events (1) | 412/1591 (26%) | 1.49 [1.23, 1.76] | 78/125 (38%) | 2.36 [1.45, 3.26] | 1.58 [0.90, 2.27] |  |
| OR^a^ (95% CI) of the psychosocial factor within genetic strata |  | 1.49 [1.23, 1.76] |  | 1.53 [0.89, 2.17] |  |  |
| High parental education (0) | 988/4994 (20%) | 1.00 (reference) | 151/537 (28%) | 1.51 [1.13, 1.90] | 1.51 [1.13, 1.90] | Multiplicative Scale:  1.07 [0.63, 1.52], p<0.001 RERI: 0.41 [-0.44, 1.26], p=0.34 |
| Low parental education (1) | 346/1285 (27%) | 1.47 [1.24, 1.70] | 58/161 (36%) | 2.40 [1.51, 3.29] | 1.63 [1.03, 2.23] |  |
| OR^a^ (95% CI) of the psychosocial factor within genetic strata |  | 1.47 [1.24, 1.70] |  | 1.58 [0.97, 2.20] |  |  |

OR = odds ratio; CI = confidence interval; PRS = polygenic risk score;

N = number of subjects

Bold values represent statistically significant OR with P < 0.05

^a^ ORs (95% CIs) were estimated by using logistic regression model with depressive symptoms (CESD≥3) as the outcome, adjusting for age, age-squared, sex, and 5 principal components. 

# **Table B. Sensitivity analyses with continuous variables.** Impact of Continuous Polygenic Score and Continuous Early Psychosocial Factors on 2006 Continuous CESD Score in 6,977 Participants

| **Variable** | **Coefficient** | **95% CI** | **p-value** |
| --- | --- | --- | --- |
| (Intercept) | 0.97 | 0.40, 1.54 | 0.00 |
| PGS MDD | 0.20 | 0.15, 0.26 | 0.00 |
| Sex | 0.40 | 0.30, 0.49 | 0.00 |
| Age | 0.00 | -0.01, 0.00 | 0.21 |
| Child Finance | 0.19 | 0.10, 0.28 | 0.00 |
| Child Stress Event | 0.28 | 0.18, 0.37 | 0.00 |
| Parental Education | 0.24 | 0.13, 0.34 | 0.00 |
| Maternal Warmth | -0.19 | -0.35, -0.04 | 0.01 |
| PC1 | -1.55 | -6.83, 3.73 | 0.57 |
| PC2 | 3.22 | -1.94, 8.38 | 0.22 |
| PC3 | 1.81 | -3.30, 6.92 | 0.49 |
| PC4 | 1.48 | -3.55, 6.50 | 0.56 |
| PC5 | -2.63 | -8.59, 3.32 | 0.39 |

# **Table C. Interaction Between Genetic Risk for Major Depressive Disorder and Cumulative Early Psychosocial Factors in Relation to CESD Score:**

| **Variables** | **Coefficient** | **95% CI** | **p-value** |
| --- | --- | --- | --- |
| Intercept | 1.00 | 0.73, 1.27 | 0.00 |
| PGS for Major Depressive Disorder | 0.20 | 0.15, 0.26 | 0.00 |
| Cumulative Early Psychosocial Experience Score | 0.12 | 0.10, 0.14 | 0.00 |
| Sex (Male vs. Female) | 0.40 | 0.30, 0.49 | 0.00 |
| Age | 0.00 | -0.01, 0.00 | 0.15 |
| PC1 | -1.40 | -6.69, 3.88 | 0.60 |
| PC2 | 2.51 | -2.58, 7.59 | 0.33 |
| PC3 | 1.76 | -3.37, 6.88 | 0.50 |
| PC4 | 1.02 | -4.03, 6.07 | 0.69 |
| PC5 | -3.20 | -9.17, 2.77 | 0.29 |
| Interaction: PGS_mdd × Cumulative Psychosocial Experience | 0.02 | 0.00, 0.04 | 0.07 |

# **Table D.** **The parental education cut point is 12 years**. Interaction of Polygenic Risk Score of Major Depressive Disorder (top 25% vs. the rest) and Early Psychosocial Experience on Depressive Symptoms (2006 CES-D Score >3) in Health and Retirement Participants (n=6,977)

| **European Americans** **(6,977)** | PRS-MDD < 75th percentile (0) |  | PRS- MDD > 75th percentile (1) |  | OR^a^ of PRS-MDD within psychosocial strata | Interaction analyses |
| --- | --- | --- | --- | --- | --- | --- |
|  | N  (cases/controls) | OR^a^ (95% CI) | N (cases/controls) | OR^a^ (95% CI) |  |  |
| High parental education (0) | 406/2500 (16%) | 1.00 (reference) | 189/763 (25%) | 1.58 [1.26, 1.91] | 1.67 [1.26, 2.08] | Multiplicative Scale:  0.97 [0.64, 1.30], p=0.84 RERI:  0.22 [-0.29, 0.73] p=0.40 |
| Low parental education - $≤$**12 years** (1) | 600/2739 (22%) | 1.44 [1.22, 1.67] | 308/975 (32%) | 2.34 [1.88, 2.80] | 1.62 [1.31, 1.92] |  |
| OR^a^ (95% CI) of the psychosocial factor within genetic strata |  | 1.44 [1.22, 1.67] |  | 1.40 [1.05, 1.75] |  |  |
| High parental education (0) | 777/4236 (18%) | 1.00 (reference) | 337/1287 (19%) | 1.55 [1.29, 1.82] | 1.55 [1.29, 1.82] | Multiplicative Scale:  1.06 [0.62, 1.50], p=0.70 RERI: 0.42 [-0.17, 1.01], p=0.16 |
| Low parental education: $≤$ **8 years** (1) | 251/1003 (25%) | 1.50 [1.23, 1.77] | 161/1451 (36%) | 2.47 [1.85, 3.10] | 1.65 [1.21, 2.09] |  |
| OR^a^ (95% CI) of the psychosocial factor within genetic strata |  | 1.50 [1.23, 1.77] |  | 1.59 [1.19, 1.99] |  |  |

OR = odds ratio; CI = confidence interval; PRS = polygenic risk score;

N = number of subjects

Bold values represent statistically significant OR with P < 0.05

^a^ ORs (95% CIs) were estimated by using logistic regression model with depressive symptoms (CESD>3) as the outcome, adjusting for age, age-squared, sex, and 5 principal components.
